# Synthesis of a Novel Series of Amino Acid Prodrugs Based on Thienopyridine Scaffolds and Evaluation of Their Antiplatelet Activity

**DOI:** 10.3390/molecules23051041

**Published:** 2018-04-28

**Authors:** Nan Lu, Lingjun Li, Xuemin Zheng, Shijun Zhang, Yuquan Li, Jing Yuan, Qunchao Wei, Youjun Xu, Fancui Meng

**Affiliations:** 1 School of Pharmaceutical Engineering, and Key Laboratory of Structure-Based Drug Design & Discovery (Ministry of Education), Shenyang Pharmaceutical University, Shenyang 110016, China; lunantj@126.com; 2 Tianjin Key Laboratory of Molecular Design and Drug Discovery, Tianjin Institute of Pharmaceutical Research, Tianjin 300193, China; lilj@tjipr.com (L.L.); zhengxm@tjipr.com (X.Z.); zhangsj@tjipr.com (S.Z.); liyq@tjipr.com(Y.L.); yuanj@tjipr.com (J.Y.); weiqc@tjipr.com (Q.W.)

**Keywords:** thienopyridines, P2Y_12_ receptor antagonist, antiplatelet, amino acid prodrugs, ADP-induced platelet aggregation

## Abstract

The thienopyridines class of drugs used as P2Y_12_ receptor antagonists plays a vital role in antiplatelet therapy. To further optimized this compound class, we designed and synthesized a series of amino acid prodrugs of 2-hydroxytetrahydrothienopyridine. All compounds were then evaluated for their inhibitory effect on ADP-induced platelet aggregation in rats and then ED_50_ and bleeding time of the most potent compounds were compared with commercial drugs. The results showed compound **5c** could be a potent and safe candidate for further research.

## 1. Introduction

**Figure 1:**
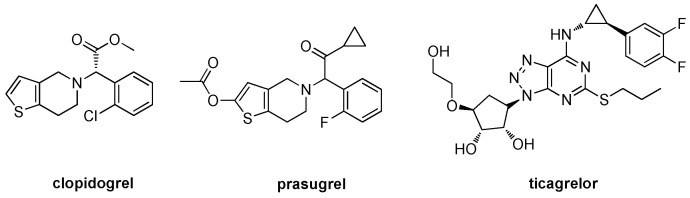
The representative chemical structures of drugs of ADP receptor antagonist.

**Figure 2:**
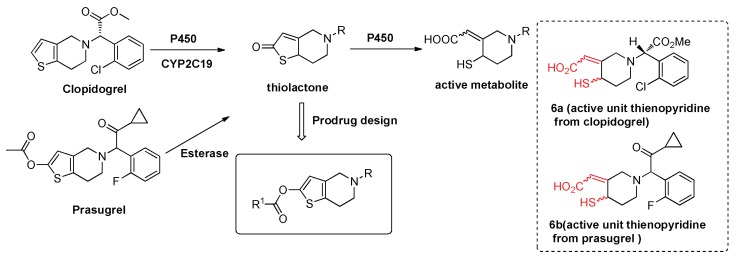
Simplified biosynthetic mechanism of the active units of thienopyridines and prodrug design.

Platelets play a critical role in the development of acute coronary syndromes (ACS) and contribute to cerebrovascular events through adhesion, aggregation and subsequent thrombus formation [1]. After platelet activation, ADP is released from intracellular storage granules and then further activates platelets, enlarging the activation and thus aggregation processes [2,3]. It was shown that the ADP response was due to activation of two receptors, the G_q_-coupled P2Y_1_ receptor, which induces a calcium response and shape change of the blood platelet and the G_i_-coupled P2Y_12_R, which decreases the intracellular adenylyl cyclase activity and prolongs intracellular calcium signaling, thereby stabilizing the formed platelet aggregates [4,5,6,7]. Consequently, blocking the P2Y_12_R is a valid strategy to antiplatelet therapy, as demonstrated by the thienopyridine class of drugs, including clopidogrel and prasugrel (Figure 1). Thienopyridines are prodrugs those are converted into their respective active metabolites (AMs) through thiolactone intermediates. Clopidogrel is oxidized by cytochrome P450 (CYP) isoforms to its thiolactone, while prasugrel is rapidly hydrolysed by esterases to its thiolactone intermediate (Figure 2) [8]. Until now, dual antiplatelet therapy with aspirin and clopidogrel has remained the treatment of choice for patients with ACS and for those undergoing percutaneous coronary interventions [9,10,11,12]. However, up to 30% of Caucasian patients carry CYP2C19 loss-of-function alleles. They cannot complete the oxidative biotransformation after receiving clopidogrel and thus are prone to suffer a high rate of subsequent cardiovascular events [13,14]. Prasugrel achieved more pronounced inhibition of platelet aggregation and lower interindividual variability of pharmacological response, but the improved efficacy was associated with an increased bleeding risk [15]. Ticagrelor, unlike the thienopyridines, is an oral cyclopentyl-triazolopyrimidine (CPTP) that is a direct and reversible inhibitor of the P2Y_12_ receptor and does not require CYP450-mediated activation. Although it effectively reduces the ischaemic events and mortality rates of cardiovascular patients, high nonlethal bleeding rates are observed and obvious undesirable side-effect appear [16]. The TRITON and PLATO clinical trials have both accounted for the long-standing hypothesis of more potent platelet inhibition translating into reduced atherothrombotic events at the expense of increased bleeding [15,16,17,18]. All these considerations reinforce the strong need of novel and safe P2Y_12_ antagonists.

**Scheme 1:**
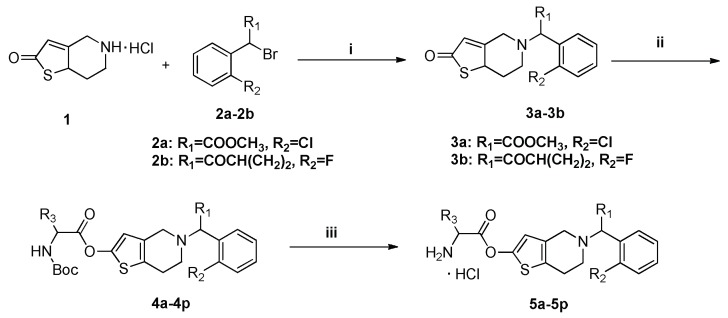
Synthetic route of compounds **5a**–**5p**. *Reagents and conditions*: (i) K_2_CO_3_, CH_3_CN, r.t. overnight (60–65% yield); (ii) *N*-Boc-l-amino acid, EDCI, DMAP, DCM, 0 °C-r.t., 4 h (71–86% yield); (iii) hydrochloric ethyl acetate, r.t., 5 h (84–93% yield).

In this study, we mimicked the metabolic pattern of prasugrel and aimed to find a drug candidate overcoming the drawbacks of clopidogrel and prasugrel, assuming that ester prodrugs might be readily converted to thiolactones by esterase-mediated hydrolysis and subsequently to the active metabolite through only one CYP-dependent step. We chose the most potential intermediates **3a** and **3b** (Scheme 1) which are, respectively, the thiolactone of clopidogrel and prasugrel, as a parent part of the target compounds.

Furthermore, amino acid prodrug design has been approached into our work as an attractive strategy. There are some good reasons for amino acid prodrugs: (1) they can be conveniently hydrolysed to their parent drug and amino acid part by enzymes in vivo. Amino acids are generally regarded as safe because they building blocks for proteins; (2) it is also proven that they can improve oral delivery and sustained release [19].

Taking the above information into account, we introduced several amino acids into the thiolactone moiety and synthesized a series of amino acid prodrugs of 2-hydroxytetra-hydrothienopyridine as novel antiplatelet agents. We also describe the inhibition of ADP-induced platelet aggregation in rats. Moreover, the potent compounds were tested for most ED_50_ and bleeding time.

## 2. Results

### 2.1. Chemistry

The synthesis of the molecules was designed and carried out as shown in Scheme 1. The thienopyridine hydrochloride **1** was reacted with substituted intermediates **2a**–**b** in the absence of potassium bicarbonate to afford the *N*-alkylated thiolactones **3a**–**b**, which were converted to **4a**–**4p** with *N*-boc-l-amino acids via EDCI and DMAP [20,21]. We chose several aliphatic and aromatic amino acids, especially including the l-proline and l-2-pyrrolidone-5-carboxylic acid with an imine and amide structure, respectively. Finally, the target amino acid esters **5a**–**5p** were obtained as hydrochloride salts after treating with hydrochloric acid in ethyl acetate for the removal of the Boc-group. All the structures are outlined in Table 1.

### 2.2. Biological Activity Evaluation

#### 2.2.1. Inhibition of ADP-induced Platelet Aggregation in Rats at a Dose of 3 mg/kg and 1 mg/kg

**Figure 3:**
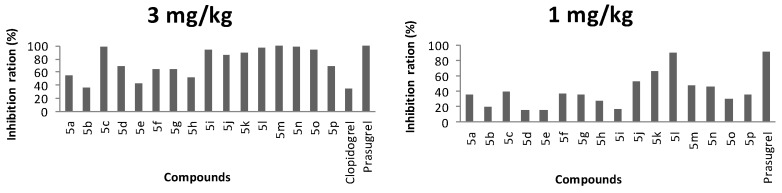
Inhibition ratios of all compounds at doses of 3 mg/kg and 1 mg/kg.

All the targeted analogues were evaluated for their inhibitory effect on ADP-induced platelet aggregation in rats, with clopidogrel and prasugrel as positive controls. The assay results are summarized in Table 2 and Figure 3. Considering that prasugrel exhibited strong potency at a dose of 3 mg/kg, while clopidogrel was almost inactive, we initiated the screening at a dose of 3 mg/kg for all compounds. As we expected, almost all the first screening round compounds presented outstanding inhibitory effect on platelet aggregation that was more potent than clopidogrel. In particular, **5c** and **5i**–**5p** showed almost equal activity to that of prasugrel at 3 mg/kg dose. To make better option for further study, we attempted to increase the difference behavior among the targeted compounds on platelet aggregation via carrying out a secondary screening of all compounds at a reduced dose of 1 mg/kg.

As shown in the 1 mg/kg level results, **5l** approached prasugrel in inhibition behavior and **5c**, **5j**, **5k**, **5m** and **5n** presented slightly less potency than prasugrel at the 1 mg/kg level. Although these compounds should covert to the same activate metabolites **6a** or **6b** to behave efficaciously [22,23], the hindrance of hydrolysis at the 2-ester moiety might have a significant impact on the potency. It was notable that **5c**, based on the 2-oxoclopidogrel structure, was superior to clopidogrel and approach to prasugrel on inhibitory of platelet aggregation.

#### 2.2.2. Determination of ED_50_ and BT_2_ of **5c** and **5l**

**Figure 4:**
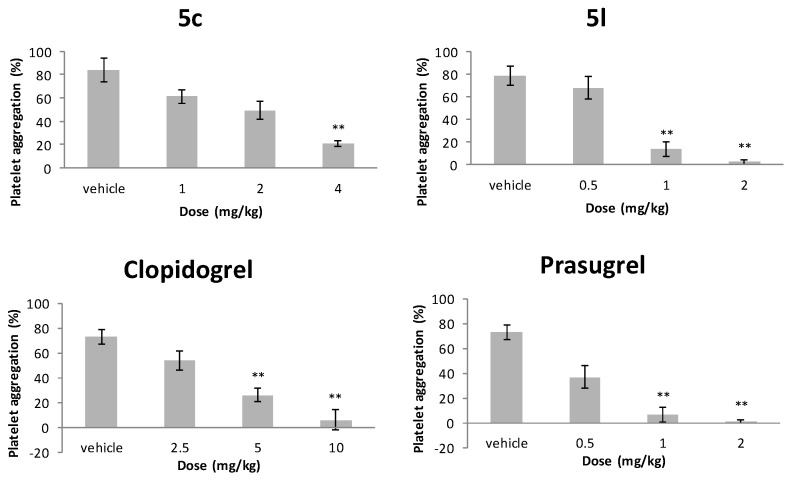
The platelet aggregation of **5c**, **5k**, clopidogrel and prasugrel at different doses. ** *p* < 0.01 vs. vehicle. Data are the mean ± SD, *n* = 5).

**Figure 5:**
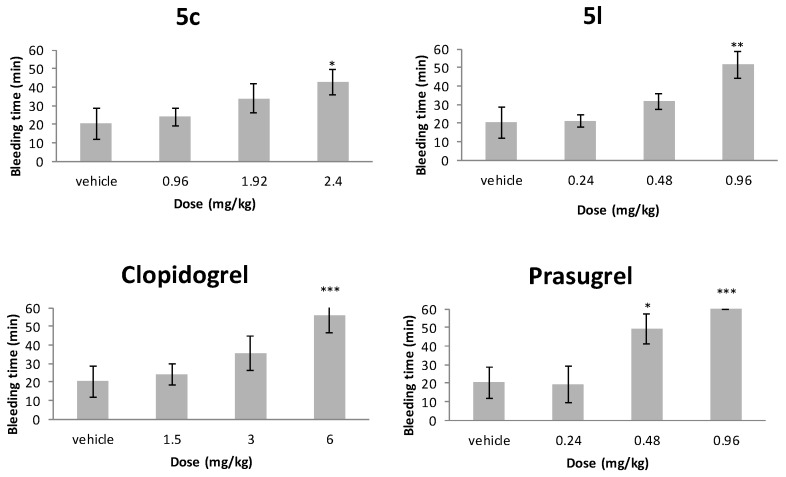
The bleeding time of **5c** and **5k** at different doses. * *p* < 0.05, ** *p* < 0.01, *** *p* < 0.001 vs. vehicle. Data are the mean ± SD, *n* = 5).

Aiming to find a candidate which has a balance between antiplatelet effects and bleeding complications, we selected **5c** and **5l** to further test for ED_50_ value and bleeding risk, because **5c** and **5l** performed the best in the aggregation assays among their own series of backbone structures which included **3a** (2-oxoclopidogrel) and **3b** (thiolactone of prasugrel), respectively. Firstly, we executed above bio-assay at doses of 0.5–4 mg/kg to determine the ED_50_ values of compounds **5c** and **5l** (Figure 4). The results showed that **5c** (ED_50_ = 2.16 mg/kg) and **5l** (ED_50_ = 0.74 mg/kg) had a moderate value between clopidogrel (ED_50_ = 3.96 mg/kg) and prasugrel (ED_50_ = 0.50 mg/kg). Moreover, in the tail bleeding test (Figure 5), **5c** induced much shorter bleeding time in rats, while **5l** showed a slightly shorter bleeding time than prasugrel. Table 3 summarizes the ED_50_ and BT_2_ values for the tested compounds. The BT_2_ was defined as the dose that doubled the vehicle bleeding time. The ED_50_ and BT_2_ values of clopidogrel and prasugrel were in good agreement with those reported [24,25]. In addition, the ratios of ED_50_ to BT_2_ indicated the tested compounds have a similar benefit/bleeding ratio risk. It should be noted that the ratio of **5c** was the lowest one, although its efficacy was lower than that of prasugrel. Taken together, these results suggest that **5c** is a potent antiplatelet agent with relatively moderate antiheamostatic potency, but this remains to be proven in future clinical studies.

## 3. Discussion

It has been reported that, at same dose, the activite metabolite of prasugrel (compound **6b**) is more potent at inhibiting platelet aggregation than that of clopidogrel (compound **6a**) [26]. This very important point is exactly conformed by our study results wherebyt compounds **5i**–**5p** generally were more effective than **5a**–**5h**, because of the generation of their respective AM after administration at the same dose. It is also indicated that **5a**–**5h** can easier and more directly transform into **3a** than clopidogrel via the evidence that **5a**–**5h** are superior to clopidogrel while **5i**–**5p** are not so to prasugrel. Taking all this into account, we believe that the compounds we designed and synthesized, especially **5a**–**5h,** are metabolized to **3a** via a one-esterase hydrolysis-step and one-P450-step in vivo, which exactly overcomes the drawback of clopidogrel resistance. Further studies will focus on the pharmacokinetics of **5c**.

## 4. Materials and Methods

### 4.1. General Information

All reagents and solvents were purchased from commercial suppliers and used without further purification. Reactions were monitored by thin layer chromatography. The melting points (m.p.) of the compounds were determined on a YRT-3 Melting Point Tester (Precision Instrument of Tianjin University, Tianjin, China). ^1^H-NMR and ^13^C-NMR spectra were recorded for DMSO-*d*_6_ solutions on a 400 MHz Bruker spectrometer (Bruker, Billerica, MA, USA). MS were measured on a Finnigan LCQ Mass (Thermo Fisher Scientific, Waltham, MA, USA), HRMS were measured on a TOF LC/MS instrument (Agilent Technologies, Santa Clara, CA, USA). Blood sample were handled with a low speed table centrifuge (LD5-2A, Beijing, China) and an aggregometer (LBY-NJ4, Beijing, China). The ED_50_ was calculated using the SPSS software (IBM, North Castle, NY, USA).

### 4.2. Chemistry

*Methyl 2-(2-chlorophenyl)-2-(2-oxo-7,7a-dihydrothieno[3,2-c]pyridin-5(2H,4H,6H)-yl)acetate* (**3a**). To a stirred solution of methyl 2-bromo-2-(2-chlorophenyl)acetate (**2a**, 26.3 g, 0.1 mol) in CH_3_CN (500 mL) were added 5,6,7,7a-tetrahydrothieno[3,2-c]pyridin-2(4*H*)-one hydrochloride (**1**, 20.9 g, 0.11 mol) and potassium bicarbonate (30.0 g, 0.3 mol). The reaction was stirred at room temperature overnight. The reaction mixture was filtered and the liquid was concentrated under reduced pressure. The residue was purified by column chromatography to give a yellow oil, which was recrystallized from EtOH to afford a white solid (20.9 g, 62% yield). m.p.: 118–119 °C. ^1^H-NMR(400 MHz, DMSO-*d*_6_): δ 7.50–7.46 (m, 2H), 7.41–7.35 (m, 2H), 6.20 (s, 1H), 4.85 (s, 1H), 4.50 (q, 1H, *J* = 6.0 Hz), 3.86 (dd, 1H, *J* = 1.2 Hz), 3.65 (s, 3H), 3.22 (d, 1H, *J* = 12.0 Hz), 2.92 (d, 1H, J = 12.0 Hz), 2.58 (t, 1H, *J* = 12.0 Hz), 2.40–2.35 (m, 1H), 1.62–1.53 (m, 1H). ESI-MS (*m*/*z*) = 338.16 [M + H]^+^. The ^1^H-NMR and MS data were in good agreement with those reported [27].

*5-(2-Cyclopropyl-1-(2-fluorophenyl)-2-oxoethyl)-5,6,7,7a-tetrahydrothieno[3,2-c]pyridin-2(4H)-one* (**3b**). Compound **3b** was synthesized from **2b** and compound **1** according to the procedure described for the preparation of **3a**. White solid product (20.0 g, 65% yield), m.p. 122–124 °C. ^1^H-NMR (400 MHz, DMSO-*d6*): δ 7.37–7.31 (m, 2H), 7.24–7.10 (m, 2H), 6.03 (s, 1H), 4.82 (s, 1H), 4.07 (q, 1H, *J* = 4.8 Hz), 3.94 (dd, 1H, *J* = 12.0 Hz), 3.08–3.05 (m, 2H), 2.35–2.30 (m, 2H), 2.12–2.06 (m, 1H), 1.93–1.83 (m, 1H), 1.04–1.01 (m, 2H), 0.90–0.78 (m, 2H). ESI-MS (*m*/*z*) = 332.19 [M + H]^+^. The ^1^H-NMR and MS data were in good agreement with those reported [20]. 

*5-(1-(2-chlorophenyl)-2-methoxy-2-oxoethyl)-4,5,6,7-tetrahydrothieno[3,2-c]pyridin-2-yl-2-((tert-butoxy-carbonyl)amino)propanoate* (**4a**). EDCI (1.2 g, 6 mmol) was slowly added to an ice-cooled mixture of **3a** (1.0 g, 3 mmol), l-*N*-Boc-alanine (0.7 g, 3.6 mmol), DMAP (40 mg, 0.3 mmol) in DCM (15 mL). The mixture was gradually warmed to room temperature and stirred for additional 4.0 h until the completion of the reaction detected by TLC. Then it was poured into ice-water (500 mL). The organic was separated and the aqueous was extracted with DCM. The combined organic was successively washed with cold 1.0 M aq HCl, saturated aq Na_2_CO_3_, and brine. It was dried over Na_2_SO_4_ and vacuum evaporated to give 4a (1.2 g, 81.2% yield) as a yellow oil. ^1^H-NMR (400 MHz, DMSO-*d*_6_): 7.55 (d, 1H, *J* =7.8 Hz), 7.50–7.45 (m, 2H), 7.38–7.32 (m, 2H), 6.40 (s, 1H), 4.82 (s, 1H), 4.21–4.18 (m, 1H), 3.63 (s, 3H), 3.52 (s, 2H), 2.83–2.74 (m, 2H), 2.66 (s, 2H), 1.45–1.30 (m, 12H). ESI-MS (*m*/*z*) = 509.08 [M + H]^+^.

Compounds **4b**–**4p** were synthesized according to the procedure described for the preparation of **4a**.

*5-(1-(2-Chlorophenyl)-2-methoxy-2-oxoethyl)-4,5,6,7-tetrahydrothieno[3,2-c]pyridin-2-yl-2-((tert-butoxy- carbonyl)-amino)-3-methylbutanoate* (**4b**). Yellow oil. ^1^H-NMR (400 MHz, DMSO-*d*_6_): δ 7.55 (d, 1H, *J* = 7.2 Hz), 7.45 (t, 2H, *J* = 8.0 Hz and 9.2 Hz), 7.38–7.31 (m, 2H), 6.40 (s, 1H), 4.81 (s, 1H), 3.99 (t, 1H, *J* = 6.8 Hz), 3.63 (s, 3H), 3.52 (s, 2H), 2.83–2.66 (m, 4H), 2.10–2.05 (m, 1H), 1.37 (s, 9H), 0.92–0.89 (m, 6H). ESI-MS (*m*/*z*) = 537.04 [M + H]^+^.

*5-(1-(2-Chlorophenyl)-2-methoxy-2-oxoethyl)-4,5,6,7-tetrahydrothieno[3,2-c]pyridin-2-yl-2-((tert-butoxy-carbonyl)amino)-4-methylpentanoate* (**4c**). Yellow oil. ^1^H-NMR (400 MHz, DMSO-*d*_6_): 7.55 (d, 1H, *J* = 6.8 Hz), 7.47 (d, 2H, *J* = 7.2 Hz), 7.39–7.32 (m, 2H), 6.40 (s, 1H), 4.83 (s, 1H), 4.16–4.10 (m, 1H), 3.63 (s, 3H), 3.52 (s, 2H), 2.83–2.60 (m, 4H), 1.65–1.40 (m, 3H), 1.36 (s, 9H), 0.88–0.84 (m, 6H). ESI-MS (*m*/*z*) = 551.05 [M + H]^+^, HRMS(ESI) calcd. for C_27_H_36_ClN_2_O_6_S^+^: [M + H]^+^ *m*/*z*: 551.1977, found: 551.1968.

*5-(1-(2-Chlorophenyl)-2-methoxy-2-oxoethyl)-4,5,6,7-tetrahydrothieno[3,2-c]pyridin-2-yl-2-((tert-butoxy-carbonyl)amino)-3-methylpentanoate* (**4d**). Yellow oil. ^1^H-NMR (400 MHz, DMSO-*d*_6_): δ 7.57 (d, 1H, *J* = 9.2 Hz), 7.48–7.34 (m, 4H), 6.42 (s, 1H), 4.83 (s, 1H), 4.08–4.04 (m, 1H), 3.65 (s, 3H), 3.53 (s, 2H), 2.85–2.75 (m, 2H), 2.65–2.63 (m, 2H), 1.88–1.80 (m, 1H), 1.38–1.18 (m, 11H), 0.88–0.73 (m, 6H). ESI-MS (*m*/*z*) = 551.10 [M + H]^+^.

*5-(1-(2-Chlorophenyl)-2-methoxy-2-oxoethyl)-4,5,6,7-tetrahydrothieno[3,2-c]pyridin-2-yl-2-((tert-butoxy-carbonyl)amino)-3-phenylpropanoate* (**4e**). Yellow oil. ^1^H-NMR (400 MHz, DMSO-*d*_6_): δ 7.38 (t, 2H, *J* = 6.4 Hz), 7.31–7.29 (m, 1H), 7.22–7.16 (m, 2H), 7.12–7.03 (m, 5H), 6.16 (s, 1H), 4.66 (s, 1H), 4.23–4.14 (m, 1H), 3.47 (s, 3H), 3.35 (s, 2H), 3.26–3.10 (m, 2H), 2.92–2.80 (m, 2H), 2.67–2.57 (m, 2H), 1.16 (s, 9H). ESI-MS (*m*/*z*) = 585.11 [M + H]^+^.

*5-(1-(2-Chlorophenyl)-2-methoxy-2-oxoethyl)-4,5,6,7-tetrahydrothieno[3,2-c]pyridin-2-yl-2-((tert-butoxy-carbonyl)amino)-3-(1H-indol-2-yl)propanoate* (**4f**). Yellow oil. ^1^H-NMR (400 MHz, DMSO-d_6_): δ 10.86 (s, 1H), 7.59 (d, 1H, *J* = 8.0 Hz), 7.53–7.48 (m, 3H), 7.39–7.33 (m, 2H), 7.18 (s, 1H), 7.06 (t, 1H, *J* = 8.0 Hz), 6.98 (t, 1H, *J* = 8.0 Hz), 6.26 (s, 1H), 4.83 (s, 1H), 4.42–4.37 (m, 1H), 3.65 (s, 3H), 3.52 (s, 2H), 3.19–3.12 (m, 2H), 2.86–2.75 (m, 2H), 2.66–2.58 (m, 2H), 1.35 (s, 9H). ESI-MS (*m*/*z*) = 624.17 [M + H]^+^.

*1-tert-Butyl 2-(5-(1-(2-chlorophenyl)-2-methoxy-2-oxoethyl)-4,5,6,7-tetrahydrothieno[3,2-c]pyridin-2-yl) pyrrolidine-1,2-dicarboxylate* (**4g**). Yellow oil. ^1^H-NMR (400 MHz, DMSO-*d*_6_): δ 7.56 (t, 1H, *J* = 2.0 Hz and 6.8 Hz), 7.46 (t, 1H, *J* = 13.6 Hz), 7.38–7.32 (m, 2H), 6.44 (s, 1H), 4.82 (s, 1H), 4.37–4.34 (m, 1H), 3.63 (s, 3H), 3.52 (s, 2H), 3.41–3.27 (m, 2H), 2.80–2.74 (m, 2H), 2.66 (s, 2H), 2.33–2.26 (m, 1H), 1.98–1.94 (m, 1H), 1.86–1.80 (m, 2H), 1.35 (s, 9H). ESI-MS (*m*/*z*) = 535.05 [M + H]^+^.

*1-tert-Butyl 2-(5-(1-(2-chlorophenyl)-2-methoxy-2-oxoethyl)-4,5,6,7-tetrahydrothieno[3,2-c]pyridin-2-yl) 5-oxopyrrolidine-1,2-dicarboxylate* (**4h**). Yellow oil. ^1^H-NMR (400 MHz, DMSO-*d*_6_): δ 7.35 (dd, 1H, *J* = 7.2 Hz), 7.28–7.25 (m, 1H), 7.17–7.14 (m, 2H), 6.30 (s, 1H), 4.66–4.62 (m, 2H), 3.43 (s, 3H), 3.22 (s, 2H), 2.60–2.47 (m, 4H), 2.30–2.18 (m, 3H), 1.85–1.76 (m, 1H), 1.17 (s, 9H). ESI-MS (*m*/*z*) = 549.05 [M + H]^+^.

*5-(2-Cyclopropyl-1-(2-fluorophenyl)-2-oxoethyl)-4,5,6,7-tetrahydrothieno[3,2-c]pyridin-2-yl-2-((tert-butoxy- carbonyl)amino)propanoate* (**4i**). Yellow oil. ^1^H-NMR (400 MHz, DMSO-*d*_6_): 7.52–7.49 (m, 1H), 7.40–7.38 (m, 2H), 7.27–7.22 (m, 2H), 6.43 (s, 1H), 4.78 (s, 1H), 4.08–4.06 (m, 1H), 3.48 (s, 2H), 2.89–2.72 (m, 4H), 2.27–2.25 (m, 1H), 1.47–1.15 (m, 12H), 0.88–0.80 (m, 4H). ESI-MS (*m*/*z*) = 503.11 [M + H]^+^.

*5-(2-Cyclopropyl-1-(2-fluorophenyl)-2-oxoethyl)-4,5,6,7-tetrahydrothieno[3,2-c]pyridin-2-yl-2-((tert-butoxy-carbonyl)amino)-3-methylbutanoate* (**4j**). White solid. m.p. 106–108 °C. ^1^H-NMR (400 MHz, DMSO-*d*_6_): δ 7.52–7.45 (m, 2H), 7.42–7.37 (m, 1H), 7.26–7.22 (m, 2H), 6.44 (s, 1H), 4.78 (s, 1H), 4.00 (t, 1H, *J* = 6.4 Hz), 3.44 (t, 2H, *J* = 16.0 Hz), 2.80–2.69 (m, 4H), 2.38–2.36 (m, 1H), 2.11–2.06 (m, 1H), 1.38 (s, 9H), 0.94–0.78 (m, 10H). ESI-MS (*m*/*z*) = 531.22 [M + H]^+^, HRMS(ESI) calcd. for C_28_H_36_FN_2_O_5_S^+^: [M + H]^+^ *m*/*z*: 531.2323, found: 531.2314.

*5-(2-Cyclopropyl-1-(2-fluorophenyl)-2-oxoethyl)-4,5,6,7-tetrahydrothieno[3,2-c]pyridin-2-yl-2-((tert-butoxy- carbonyl)amino)-4-methylpentanoate* (**4k**). Yellow oil. ^1^H-NMR (400 MHz, DMSO-*d*_6_): 7.52–7.48 (m, 2H), 7.40 (dd, 1H, *J* = 13.6 Hz), 7.26–7.21 (m, 2H), 6.43 (s, 1H), 4.78 (s, 1H), 4.17–4.12 (m, 1H), 3.44 (t, 2H, *J* = 6.4 Hz), 2.80–2.70 (m, 4H), 2.37–2.35 (m, 1H), 1.66–1.42 (m, 3H), 1.37 (s, 9H), 0.90–0.80 (m, 10H). ESI-MS (*m*/*z*) = 545.14 [M + H]^+^.

*5-(2-Cyclopropyl-1-(2-fluorophenyl)-2-oxoethyl)-4,5,6,7-tetrahydrothieno[3,2-c]pyridin-2-yl-2-((tert-butoxy-carbonyl)amino)-3-methylpentanoate* (**4l**). White solid. m.p.: 80–82 °C. ^1^H-NMR (400 MHz, DMSO-*d*_6_): δ 7.52–7.45 (m, 2H), 7.40 (dd, 1H, *J* = 12.8 Hz), 7.26–7.21 (m, 2H), 6.43 (s, 1H), 4.77 (s, 1H), 4.05 (t, 1H, *J* = 6.8 Hz), 3.44 (t, 2H, *J* = 15.2Hz), 2.80–2.69 (m, 4H), 2.39–2.35 (m, 1H), 1.86–1.82 (m, 1H), 1.38–1.20 (m, 11H), 0.88–0.73 (m, 10H). ESI-MS (*m*/*z*) = 545.10 [M + H]^+^, HRMS(ESI) calcd. for C_29_H_38_FN_2_O_5_S^+^: [M + H]^+^ *m*/*z*: 545.2480, found: 545.2467.

*5-(2-Cyclopropyl-1-(2-fluorophenyl)-2-oxoethyl)-4,5,6,7-tetrahydrothieno[3,2-c]pyridin-2-yl-2-((tert-butoxy-carbonyl)amino)-3-phenylpropanoate* (**4m**). White solid. m.p.: 110–112 °C. ^1^H-NMR (400 MHz, DMSO-*d*_6_): δ 7.54 (d, 1H, *J* = 7.2 Hz), 7.48 (t, 1H, *J* = 7.2 Hz), 7.40–7.36 (m, 1H), 7.28–7.20 (m, 7 H), 6.32 (s, 1H), 4.76 (s, 1H), 4.35–4.14 (m, 1H), 3.45–3.36 (m, 2H), 3.05–3.00 (m, 2H), 2.79–2.66 (m, 4H), 2.36–2.34 (m, 1H), 1.31 (s, 9H), 0.87–0.76 (m, 4H). ESI-MS (*m*/*z*) = 579.17 [M+H]^+^, HRMS(ESI) calcd. for C_32_H_36_FN_2_O_5_S^+^: [M + H]^+^ *m*/*z*: 579.2323, found: 579.2315.

*5-(2-Cyclopropyl-1-(2-fluorophenyl)-2-oxoethyl)-4,5,6,7-tetrahydrothieno[3,2-c]pyridin-2-yl-2-((tertbutoxy- carbonyl)amino)-3-(1H-indol-2-yl)propanoate* (**4n**). Yellow oil. ^1^H-NMR (400 MHz, DMSO-*d*_6_): δ 10.86 (s, 1H), 7.52–7.49 (m, 3H), 7.40 (dd, 1H, *J* = 13.2Hz), 7.33 (d, 1H, *J* = 8.0 Hz), 7.27–7.22 (m, 2H), 7.18 (s, 1H), 7.08–6.95 (m, 2H), 6.26 (s, 1H), 4.78 (s, 1H), 4.40–4.36 (m, 1H), 3.45–3.37 (m, 2H), 3.18–3.14 (m, 2H), 2.80–2.68 (m, 4H), 2.38–2.36 (m, 1H), 1.34 (s, 9H), 0.88–0.80 (m, 4H). ESI-MS (*m*/*z*) = 618.23 [M + H]^+^.

*1-tert-Butyl 2-(5-(2-cyclopropyl-1-(2-fluorophenyl)-2-oxoethyl)-4,5,6,7-tetrahydrothieno[3,2-c]pyridin-2-yl) pyrrolidine-1,2-dicarboxylate* (**4o**). Yellow oil. ^1^H-NMR (400 MHz, DMSO-*d*_6_): δ 7.50, (t, 1H, *J* = 7.2 Hz), 7.40 (dd, 1H, *J* = 13.6 Hz), 7.28–7.22 (m, 2H), 6.47 (s, 1H), 4.78 (s, 1H), 4.42–4.36 (m, 1H), 3.65 (m, 1H), 3.48–3.33 (m, 4H), 3.03–2.79 (m, 4H), 2.37–2.24 (m, 2H), 2.02–2.00 (m, 1H), 1.98–1.87 (m, 2H), 1.39 (s, 9H), 0.88–0.75 (m, 4H). ESI-MS (*m*/*z*) = 529.10 [M + H]^+^.

*1-tert-Butyl 2-(5-(2-cyclopropyl-1-(2-fluorophenyl)-2-oxoethyl)-4,5,6,7-tetrahydrothieno[3,2-c]pyridin-2-yl) 5-oxopyrrolidine-1,2-dicarboxylate* (**4p**). Yellow oil. ^1^H-NMR (400 MHz, DMSO-*d*_6_): δ 7.50 (t, 1H, *J* = 7.2 Hz), 7.40 (dd, 1H, *J* = 13.2Hz), 7.27–7.22 (m, 2H), 6.53 (s, 1H), 4.87(q, 1H, *J* = 5.6 HZ), 4.79 (s, 1H), 3.49–3.41 (m, 2H), 2.82–2.69 (m, 4H), 2.56–2.34 (m, 4H), 2.08–2.01 (m, 1H), 1.39 (s, 9H), 0.88–0.84 (m, 4H). ESI-MS (*m*/*z*) = 543.15 [M + H]^+^.

*5-(1-(2-Chlorophenyl)-2-methoxy-2-oxoethyl)-4,5,6,7-tetrahydrothieno[3,2-c]pyridin-2-yl 2-aminopropanoate hydrochloride* (**5a**). Compound **4a** (1.0 g, 2.0 mmol) was stirred with hydrochloric ethyl acetate (10 mL, 2.0 M) at r.t. for 5.0 h. The formed precipitate was filtered and dried to give compound **5a** (0.82 g, 92.1%) as a white solid. m.p.: 149−151 °C. ^1^H-NMR (400 MHz, DMSO-*d*_6_): δ 8.80 (s, 3H), 7.57–7.55 (m, 1H), 7.52–7.40 (m, 3H), 6.62 (s, 1H), 5.30 (m, 1H), 4.38 (s, 1H), 3.93 (s, 2H), 3.70 (s, 3H), 3.20–2.88 (m, 4H), 1.52 (d, 3H, *J* = 8.0 Hz). ESI-MS (*m*/*z*) = 408.98 [M + H]^+^, HRMS(ESI) calcd. for C_19_H_22_ClN_2_O_4_S^+^: [M + H]^+^ *m*/*z*: 409.0983, found: 409.0986.

Compounds **5b**–**5p** were synthesized according to the procedure described for the preparation of **5a**.

*5-(1-(2-Chlorophenyl)-2-methoxy-2-oxoethyl)-4,5,6,7-tetrahydrothieno[3,2-c]pyridin-2-yl-2-amino-3-methyl-butanoate hydrochloride* (**5b**). White solid. m.p.: 165–167 °C. ^1^H-NMR (400 MHz, DMSO-*d*_6_): δ 8.93 (s, 3H), 7.77 (s, 1H), 7.63–7.58 (m, 1H), 7.52–7.46 (m, 2H), 6.64 (s, 1H), 5.34 (m, 1H), 4.15 (s, 1H), 3.93 (s, 2H), 3.70 (s, 3H), 3.23–2.92 (m, 4H), 2.34–2.29 (m, 1H), 1.05–0.98 (m, 6 H). ESI-MS (*m*/*z*) = 437.02 [M + H]^+^, HRMS(ESI) calcd. for C_21_H_26_ClN_2_O_4_S^+^: [M + H]^+^ *m*/*z*: 437.1296, found: 437.1333.

*5-(1-(2-Chlorophenyl)-2-methoxy-2-oxoethyl)-4,5,6,7-tetrahydrothieno[3,2-c]pyridin-2-yl-2-amino-4-methyl-pentanoate hydrochloride* (**5c**). White solid. m.p.: 133–135 °C. ^1^H-NMR (400 MHz, DMSO-*d*_6_): δ 8.82 (s, 3H), 7.69–7.64 (m, 1H), 7.62–7.59 (m, 1H), 7.51–7.40 (m, 2H), 6.62 (s, 1H), 5.20 (m, 1H), 4.26 (s, 1H), 3.89 (s, 2H), 3.70 (s, 3H), 3.12–2.86 (m, 4H), 2.70–2.66 (m, 2H), 1.63–1.59 (m, 1H), 0.92–0.90 (m, 6 H). ESI-MS (*m*/*z*) = 451.03 [M + H]^+^, HRMS(ESI) calcd. for C_22_H_28_ClN_2_O_4_S^+^: [M + H]^+^ *m*/*z*: 451.1453, found: 451.1455. HPLC purity: 94.00%.

*5-(1-(2-Chlorophenyl)-2-methoxy-2-oxoethyl)-4,5,6,7-tetrahydrothieno[3,2-c]pyridin-2-yl-2-amino-3-methyl-pentanoate hydrochloride* (**5d**). White solid. m.p.: 150–153 °C. ^1^H-NMR (400 MHz, DMSO-*d*_6_): δ 8.91 (s, 3H), 7.75–7.72 (m, 1H), 7.64–7.62 (m, 1H), 7.56–7.46 (m, 2H), 6.63 (s, 1H), 5.30–5.26 (m, 1H), 4.21 (s, 1H), 3.87 (s, 2H), 3.70 (s, 3H), 3.17–2.88 (m, 4H), 2.08–1.99 (m, 1H), 1.56–1.49 (m, 1H), 1.36–1.29 (m, 1H), 0.94–0.87 (m, 6 H). ESI-MS (*m*/*z*) = 451.04 [M + H]^+^, HRMS(ESI) calcd. for C_22_H_28_ClN_2_O_4_S^+^: [M + H]^+^ *m*/*z*: 451.1453, found: 451.1459.

*5-(1-(2-Chlorophenyl)-2-methoxy-2-oxoethyl)-4,5,6,7-tetrahydrothieno[3,2-c]pyridin-2-yl-2-amino-3-phenyl-propanoate hydrochloride* (**5e**). White solid. m.p.: 166–168 °C. ^1^H-NMR (400 MHz, DMSO-*d*_6_): δ 9.01 (s, 3H), 7.65–7.57 (m, 3H), 7.46 (d, 2H, *J* = 6.4 Hz), 7.33–7.27 (m, 4H), 6.43 (s, 1H), 5.30–5.25 (m, 1H), 4.54 (s, 1H), 3.89 (s, 2H), 3.70 (s, 3H), 3.39–3.16 (m, 2H), 3.14–2.87 (m, 4H). ESI-MS (*m*/*z*) = 485.03 [M + H]^+^, HRMS(ESI) calcd. for C_25_H_26_ClN_2_O_4_S^+^: [M + H]^+^ *m*/*z*: 485.1296, found: 485.1296.

*5-(1-(2-Chlorophenyl)-2-methoxy-2-oxoethyl)-4,5,6,7-tetrahydrothieno[3,2-c]pyridin-2-yl-2-amino-3-(1H-indol-2-yl)propanoate hydrochloride* (**5f**). White solid. m.p.: 173–175 °C. ^1^H-NMR (400 MHz, DMSO-*d*_6_): δ 11.10 (s, 1H), 8.92 (s, 3H), 7.57 (d, 2H, *J* = 7.2 Hz), 7.52–7.50 (m, 3H), 7.38 (d, 1H, *J* = 6.4 Hz), 7.25 (s, 1H), 7.08 (t, 1H, *J* = 12.0 Hz), 6.98 (t, 1H, *J* = 16.0 Hz), 6.36 (s, 1H), 5.28–5.24 (m, 1H), 4.50 (s, 1H), 3.93 (s, 2H), 3.70 (s, 3H), 3.51–3.46 (m, 1H), 3.38–3.33 (m, 1H), 3.20–2.88 (m, 4H). ESI-MS (*m*/*z*) = 524.09 [M + H]^+^, HRMS(ESI) calcd. for C_27_H_27_ClN_3_O_4_S^+^: [M + H]^+^ *m*/*z*: 524.1405, found: 524.1408.

*5-(1-(2-Chlorophenyl)-2-methoxy-2-oxoethyl)-4,5,6,7-tetrahydrothieno[3,2-c]pyridin-2-yl-pyrrolidine-2-carboxylate hydrochloride* (**5g**). White solid. m.p.: 102–104 °C. ^1^H-NMR (400 MHz, DMSO-*d*_6_): δ 10.47 (s, 1H), 9.47 (s, 1H), 7.72 (d, 1H, *J* = 8.8 Hz), 7.62–7.52 (m, 1H), 7.41–7.39 (m, 2H), 6.63 (s, 1H), 5.29–5.24 (m, 1H), 4.64 (s, 1H), 3.88 (s, 2H), 3.70 (s, 3H), 3.23–3.20 (m, 4H), 2.90–2.88 (m, 2H), 2.38–2.29 (m, 1H), 2.17–2.10 (m,1H), 1.97–1.90 (m, 2H). ESI-MS (*m*/*z*) = 434.96 [M + H]^+^, HRMS(ESI) calcd. for C_21_H_24_ClN_2_O_4_S^+^: [M + H]^+^ *m*/*z*: 435.1140, found: 435.1147.

*5-(1-(2-Chlorophenyl)-2-methoxy-2-oxoethyl)-4,5,6,7-tetrahydrothieno[3,2-c]pyridin-2-yl-5-oxopyrrolidine-2-carboxylate hydrochloride* (**5h**). White solid. m.p.: 88–90 °C. ^1^H-NMR (400 MHz, DMSO-*d*_6_): δ 8.10 (s, 1H), 7.76 (s, 1H), 7.61–7.59 (m, 1H), 7.51–7.39 (m, 3H), 6.59 (s, 1H), 5.42–5.37 (m, 1H), 4.49–4.46 (m, 1H), 3.71 (s, 3H), 3.67 (s, 2H), 3.04–2.90 (m, 4H), 2.47–2.10 (m, 4H). ESI-MS (*m*/*z*) = 449.00 [M + H]^+^, HRMS(ESI) calcd. for C_21_H_22_ClN_2_O_5_S^+^: [M + H]^+^ *m*/*z*: 449.0932, found: 449.0932.

*5-(2-Cyclopropyl-1-(2-fluorophenyl)-2-oxoethyl)-4,5,6,7-tetrahydrothieno[3,2-c]pyridin-2-yl-2-amino-propanoate hydrochloride* (**5i**). White solid. m.p.: 148–150 °C. ^1^H-NMR (400 MHz, DMSO-*d*_6_): δ 8.90 (s, 3H), 7.66–7.55 (m, 2H), 7.50–7.33 (m, 2H), 6.67 (s, 1H), 6.08–6.03 (m, 1H), 4.38 (s, 1H), 3.99 (s, 2H), 3.61–3.57 (m, 2H), 3.10–2.91 (m, 2H), 1.98–1.89 (m, 1H), 1.50 (d, 3H, *J* = 17.2 Hz), 1.07–0.93 (m, 4H). ESI-MS (*m*/*z*) = 403.05 [M + H]^+^, HRMS(ESI) calcd. for C_21_H_24_FN_2_O_3_S^+^: [M + H]^+^ *m*/*z*: 403.1486, found: 403.1491.

*5-(2-Cyclopropyl-1-(2-fluorophenyl)-2-oxoethyl)-4,5,6,7-tetrahydrothieno[3,2-c]pyridin-2-yl-2-amino-3-methylbutanoate hydrochloride* (**5j**). White solid. m.p.: 147–148 °C. ^1^H-NMR (400 MHz, DMSO-*d*_6_): δ 8.90 (s, 3H), 7.62 (d, 2H, *J* =6.4 Hz), 7.45–7.36 (m, 2H), 6.69 (s, 1H), 6.01–5.97 (m, 1H), 4.16 (s, 1H), 3.96 (s, 2H), 3.65–3.49 (m, 2H), 3.04–3.00 (m, 2H), 2.34–2.26 (m, 1H), 1.97–1.93 (m, 1H), 1.16–0.90 (m, 10H). ESI-MS (*m*/*z*) = 431.09 [M + H]^+^, HRMS(ESI) calcd. for C_23_H_28_FN_2_O_3_S^+^: [M + H]^+^ *m*/*z*: 431.1799, found: 431.1798.

*5-(2-Cyclopropyl-1-(2-fluorophenyl)-2-oxoethyl)-4,5,6,7-tetrahydrothieno[3,2-c]pyridin-2-yl-2-amino-4-methylpentanoate hydrochloride* (**5k**). White solid. m.p.: 141–143 °C. ^1^H-NMR (400 MHz, DMSO-*d*_6_): δ 8.64 (s, 3H), 7.70–7.62 (m, 2H), 7.47–7.37 (m, 2H), 6.68 (s, 1H), 6.08–6.01 (m, 1H), 4.23 (s, 1H), 3.98 (s, 2H), 3.61–3.48 (m, 2H), 3.05–3.00 (m, 2H), 2.76–2.71 (m, 2H), 1.97–1.87 (m, 1H), 1.65–1.63 (m, 1H), 0.91–0.90 (m, 10H). ESI-MS (*m*/*z*) = 445.08 [M + H]^+^, HRMS(ESI) calcd. for C_24_H_30_FN_2_O_3_S^+^: [M + H]^+^ *m*/*z*: 445.1956, found: 445.1958.

*5-(2-Cyclopropyl-1-(2-fluorophenyl)-2-oxoethyl)-4,5,6,7-tetrahydrothieno[3,2-c]pyridin-2-yl-2-amino-3-methylpentanoate hydrochloride* (**5l**). White solid. m.p.: 142–144 °C. ^1^H-NMR (400 MHz, DMSO-*d*_6_): δ 8.90 (s, 3H), 7.62–7.60 (m, 2H), 7.45–7.36 (m, 2H), 6.68 (s, 1H), 6.08–6.03 (m, 1H), 4.22 (s, 1H), 3.85 (s, 2H), 3.15–2.92 (m, 4H), 2.06–1.88 (m, 2H), 1.54–1.49 (m, 1H),1.36–1.32 (m, 1H), 1.03–0.87 (m, 10H). ESI-MS (*m*/*z*) = 445.07 [M + H]^+^, HRMS(ESI) calcd. for C_24_H_30_FN_2_O_3_S^+^: [M + H]^+^ *m*/*z*: 445.1956, found: 445.1957.

*5-(2-Cyclopropyl-1-(2-fluorophenyl)-2-oxoethyl)-4,5,6,7-tetrahydrothieno[3,2-c]pyridin-2-yl-2-amino-3-phenylpropanoate hydrochloride* (**5m**). White solid. m.p.: 156–158 °C. ^1^H-NMR (400 MHz, DMSO-*d*_6_): δ 9.06 (s, 3H), 7.65–7.63 (m, 2H), 7.43–7.28 (m, 7 H), 6.48 (s, 1H), 6.06–6.02 (m, 1H), 4.53 (s, 1H), 3.88 (s, 2H), 3.39–3.15 (m, 2H), 3.14–2.87 (m, 4H), 1.99–1.95 (m, 1H), 0.91–0.89 (m, 4H). ESI-MS (*m*/*z*) = 479.05 [M + H]^+^, HRMS(ESI) calcd. for C_27_H_28_FN_2_O_3_S^+^: [M + H]^+^ *m*/*z*: 479.1799, found: 479.1798.

*5-(2-Cyclopropyl-1-(2-fluorophenyl)-2-oxoethyl)-4,5,6,7-tetrahydrothieno[3,2-c]pyridin-2-yl-2-amino-3-(1H-indol-2-yl)propanoate hydrochloride* (**5n**). White solid. m.p.: 166–168 °C. ^1^H-NMR (400 MHz, DMSO-d6): δ 11.11 (s, 1H), 8.93 (s, 3H), 7.61–7.56 (m, 3H), 7.46–7.30 (m, 3H), 7.25 (s, 1H), 7.07 (t, 1H, J = 7.6 Hz), 6.99 (t, 1H, *J* = 7.2 Hz), 6.43 (s, 1H), 4.97–4.95 (m, 1H), 4.50 (s, 1H), 4.00 (s, 2H), 3.51–3.23 (m, 2H), 3.20–2.88 (m, 4H), 1.91–1.89 (m, 1H), 1.04–0.89 (m, 4H). ESI-MS (*m*/*z*) = 518.09 [M + H]^+^, HRMS(ESI) calcd. for C_29_H_29_FN_3_O_3_S^+^: [M + H]^+^ *m*/*z*: 518.1908, found: 518.1912.

*5-(2-Cyclopropyl-1-(2-fluorophenyl)-2-oxoethyl)-4,5,6,7-tetrahydrothieno[3,2-c]pyridin-2-yl-pyrrolidine-2-carboxylate hydrochloride* (**5o**). White solid. m.p.: 96–98 °C. ^1^H-NMR (400 MHz, DMSO-*d*_6_): δ 10.74 (s, 1H), 9.72 (s, 1H), 7.69–7.62 (m, 2H), 7.47–7.37 (m, 2H), 6.69 (s, 1H), 6.10–6.03 (m, 1H), 4.63 (s, 1H), 4.23–4.19 (s, 2H), 3.57–3.47 (m, 2H), 3.28–3.06 (m, 4H), 2.34–2.10 (m,2H), 1.97–1.90 (m, 3H), 1.07–0.90 (m, 4H). ESI-MS (*m*/*z*) = 429.08 [M + H]^+^, HRMS(ESI) calcd. for C_23_H_26_FN_2_O_3_S^+^: [M + H]^+^ *m*/*z*: 429.1643, found: 429.1640.

*5-(2-Cyclopropyl-1-(2-fluorophenyl)-2-oxoethyl)-4,5,6,7-tetrahydrothieno[3,2-c]pyridin-2-yl-5-oxo-pyrrolidine-2-carboxylate hydrochloride* (**5p**). White solid. m.p.: 98–101 °C. ^1^H-NMR (400 MHz, DMSO-*d*_6_): δ 8.11 (s, 1H), 7.64–7.39 (m, 5 H), 6.63 (s, 1H), 6.09–6.03 (m, 1H), 4.48–4.47 (m, 1H), 3.44 (s, 2H), 3.04 (m, 2H), 2.44–2.40 (m, 1H), 2.20–2.12 (m, 3H), 1.97–1.90 (m, 3H), 1.05–0.90 (m, 4H). ESI-MS (*m*/*z*) = 443.06 [M + H]^+^, HRMS(ESI) calcd. for C_23_H_24_FN_2_O_4_S^+^: [M + H]^+^ *m*/*z*: 443.1435, found: 443.1433.

### 4.3. Inhibition of ADP-Induced Platelet Aggregation in Rats

ADP-induced platelet aggregation was determined by Born’s method [28]. Male SD rats (200–300 g, 5 in each group) were orally gavaged at random with vehicle control, target compounds, clopidogrel and prasugrel. The volume of target compounds and positive control was 10 mL/kg × body weight, while the vehicle group was equal volume as experimental group instead of 0.5% CMC-Na. Two hours after administration, the animals were anesthetized (with 0.7% chloral hydrate intraperitoneal injection) and bloods were collected via aorta ventralis puncture into anticoagulant solution (3.8% sodium citrate). Platelet rich plasma (PRP) was centrifuged at 230 rpm for 15 mins and then adjusted by platelet poor plasma (PPP, centrifuged at 2000 rpm for 10 mins). Platelet count was 5 × 10^8^/mL. Aggregation was induced by ADP (20 μM) and measured using an aggregometer (LBY-NJ4, Beijing, China ). The platelet aggregation was observed maximum platelet aggregation (MPA) from the aggregometer. The percentage of inhibition of platelet aggregation (IPA) was calculated from the observed MPA by the following equation:

IPA (%) = (MPA_vehicle_ − MPA_compound_)/MPA_vehicle_ × 100%

Inhibition rats at different doses were calculated ED_50_ by the software named Statistical Product and Service Solutions (SPSS). The animal laboratory got animal use certificate issued by Science and Technology department of Tianjin (SYXK(jin)2016-0013).

### 4.4. Determination of Bleeding Time 

The tail transection bleeding time was determined by the method of Dejana et al. [29]. Male SD rats (200–300 g, 5 in each group) were orally gavaged at random with vehicle control, target compounds, clopidogrel and prasugrel. The volume of target compounds and positive control was 10 mL/kg × body weight, while the vehicle group was equal volume as experimental group instead of 0.5% CMC-Na. The test drugs were orally administered 1H before the tail transection. Under anaesthesia with urethane (5 mL/kg), the rat tail was transected at 5 mm from the tip by a scalpel, and the tail was immediately immersed into warmed (37 °C) saline until blood flow stopped. Bleeding time was assessed as the time from the tail transection to the termination of blood flow. Bleeding times beyond 60 min were regarded as 60 min for the purpose of statistical analysis. BT_2_ values were calculated from linear-regression analysis.

## 5. Conclusions

In summary, we designed and synthesized a series of amino acid prodrugs based on thienopyridine scaffolds as novel potent P2Y_12_R inhibitors. Inhibition of ADP-induced platelet aggregation in rat assays showed that compounds **5k**, **5l**, **5c**, **5j**, **5m**, and **5n** displayed good activity and compounds **5c** and **5l** have moderate ED_50_ values between those of clopidogrel and prasugrel. In the tail bleeding test, **5c** induced much shorter bleeding time in rats and **5l** behaved slightly better than prasugrel. Based on their ratio of benefit/bleeding risk, we will take **5c** as a drug candidate for further research.

## Figures and Tables

**Table 1:**
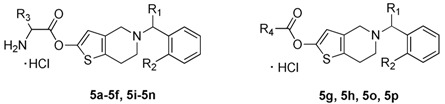
Structures of target compounds.

Compound	R_3_ or R_4_^a^ (R_1_=COOCH_3_, R_2_=Cl)	Compound	R_3_ or R_4_^a^ (R_1_=COCH(CH_2_)_2_, R_2_=F)
**5a**	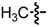	**5i**	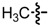
**5b**	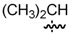	**5j**	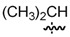
**5c**	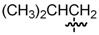	**5k**	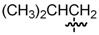
**5d**	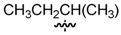	**5l**	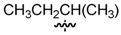
**5e**	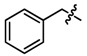	**5m**	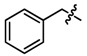
**5f**	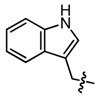	**5n**	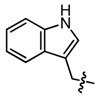
**5g**	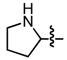	**5o**	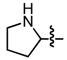
**5h**	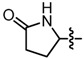	**5p**	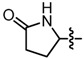

^a^ R_4_ was only used for compounds **5g**–**5h** and **5o**–**5p**.

**Table 2:** Inhibitory effect of target compounds on ADP-induced platelet aggregation in rats at a dose of 3 mg/kg and 1mg/kg.

Compounds	Inhibition Ration (%)	
	3 mg/kg	1 mg/kg
**5a**	55.1	35.1
**5b**	36.8	19.4
**5c**	98.4	39.6
**5d**	68.9	16.2
**5e**	43.3	15.8
**5f**	63.8	37.2
**5g**	64.2	35.5
**5h**	51.6	27.2
**5i**	93.4	17.5
**5j**	86.3	52.6
**5k**	89.2	66.2
**5l**	96.4	89.8
**5m**	100	47.6
**5n**	99.3	46.4
**5o**	93.6	30.7
**5p**	69.5	35.1
**Clopidogrel**	34.4	--
**Prasugrel**	100	91.0

**Table 3:** Comparison of antiplatelet antihaemostatic effects of potent compounds.

Compounds	ED_50_ (mg/kg)	BT_2_ (mg/kg)	ED_50_/BT_2_
**Clopidogrel**	3.96	3.82	1.04
**Prasugrel**	0.50	0.51	0.98
**5c**	2.16	2.32	0.93
**5l**	0.74	0.70	1.06

## Supplementary Materials

Supplementary File 1
