# Supplementary material for: Synthesis of a Novel Series of Amino Acid Prodrugs Based on Thienopyridine Scaffolds and Evaluation of Their Antiplatelet Activity
Source: Molecules. 2018 Apr 28;23(5):1041. doi: 10.3390/molecules23051041 (PMC6102589; doi:10.3390/molecules23051041)
Supplement: Supplementary File 1 [file molecules-23-01041-s001.pdf]

A novel series of amino acid prodrugs synthesized base on thienopyridines scaffolds and evaluation of their antiplatelet activity

Nan Lu[1, 2], Lingjun Li[2], Xuemin Zheng[2], Shijun Zhang[2], Yuquan Li[2], Jing Yuan[2], Qunchao Wei[2], Youjun Xu[1*], Fancui Meng[2*]

1 School of Pharmaceutical Engineering, and Key Laboratory of Structure-Based Drug Design & Discovery (Ministry of Education), Shenyang Pharmaceutical University, Shenyang 110016, China; lunantj@126.com (N. L.)

2 Tianjin Key Laboratory of Molecular Design and Drug Discovery, Tianjin Institute of Pharmaceutical Research, Tianjin 300193, China; lilj@tjipr.com (L.L.); zhengxm@tjipr.com (X.Z.); zhangsj@tjipr.com (S.Z.); liyq@tjipr.com(Y.L.); yuanj@tjipr.com (J.Y.); weiqc@tjipr.com (Q.W.)

* Correspondence: xuyoujun@syphu.edu.cn (Y. X.); mengfc@tjipr.com (F.M.); Tel.: +86-24-4352-0223(Y.X.); +86-22-2300-6833 (F.M.)

Supplementary Information

1. Platelet aggregation of target compounds

Supplementary Table 1. Platelet aggregation of first round screening target compounds at a dose of 3 mg/kg

Compounds	Platelet aggregation (%)
5a	33.4±19.3
5b	47.0±11.3
5c	1.2±2.0**
5d	23.1±6.1*
5e	42.2±16.5
5f	26.9±16.0*
5g	26.6±12.1*
5j	10.2±13.1**
5m	0±0**
clopidogrel	48.8±15.6
prasugrel	0.0±0.1**
vehicle	74.4±12.0

(*) P < 0.05 (**) P < 0.01 vs vehicle. Data are the mean ± SD, n = 5.

Supplementary Table 2. Platelet aggregation of second round screening target compounds at a dose of 3 mg/kg

Compounds	Platelet aggregation (%)
5h	27.9±7.3*
5i	3.8±1.8**
5k	6.2±7.8**
5l	2.1±3.7**
5n	0.4±0.6**
5o	3.7±7.4**

5p	17.6±9.1*
vehicle	57.7±5.7

(*) P < 0.05 (**) P < 0.01 vs vehicle. Data are the mean ± SD, n = 5.

Supplementary Table 3. Platelet aggregation of second round screening target compounds at a dose of 1 mg/kg

Compounds	platelet aggregation (%)
5a	49.1±20.7
5b	61.0±10.7
5c	45.7±11.0
5d	63.4±20.4
5e	63.7±17.2
5f	47.5±6.2
5g	48.8±18.0
5h	55.1±15.7
5i	62.5±20.0
5j	35.8±14.1*
5k	25.6±16.1*
5l	7.8±13.1*
5m	39.7±14.8*
5n	40.6±5.8
5o	52.5±11.9
5p	49.2±14.2
prasugrel	6.8±2.0**
vehicle	75.7±11.7

(*) P < 0.05 (**) P < 0.01 vs vehicle. Data are the mean ± SD, n = 5.

2. Characterization of Compound **5c** and its intermediate **4c**

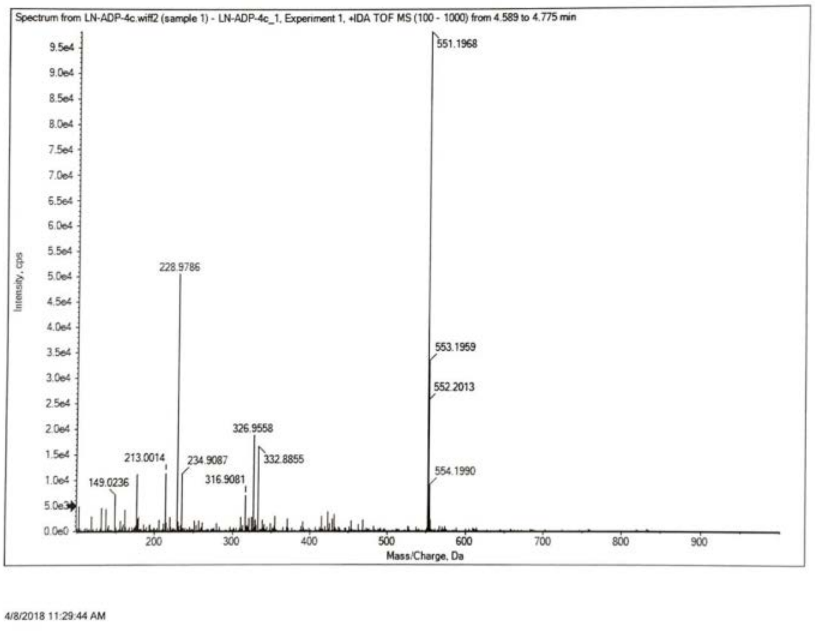

Supplementary Figure 1. HRMS of **4c**.

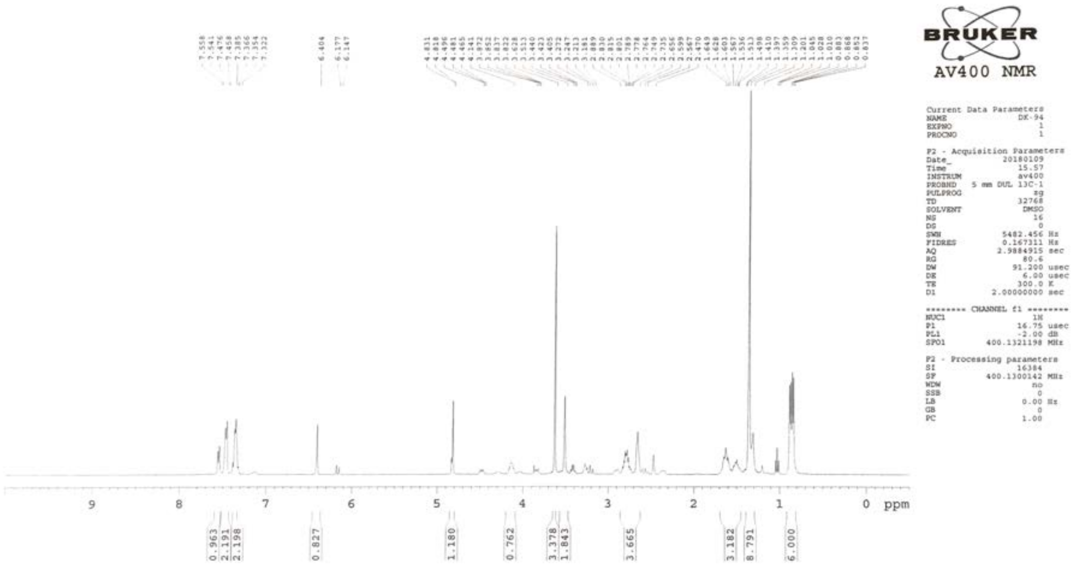

Supplementary Figure 2. ^{1}H-NMR of **4c**.

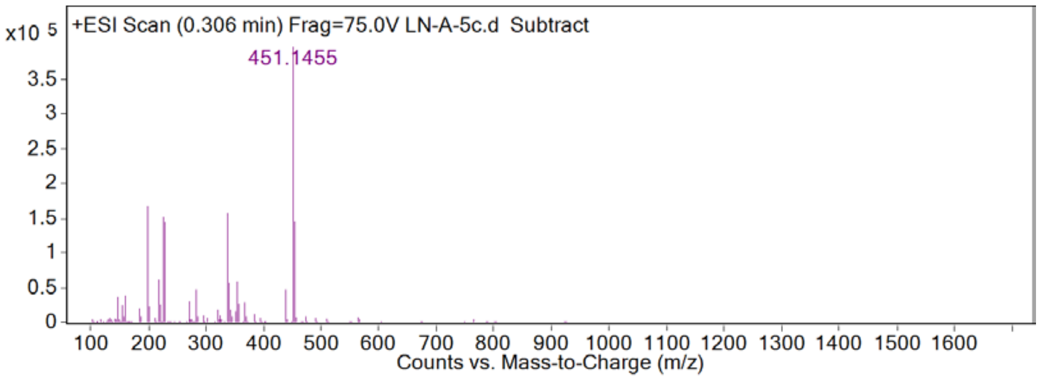

Supplementary Figure 3. HRMS of **4c**.

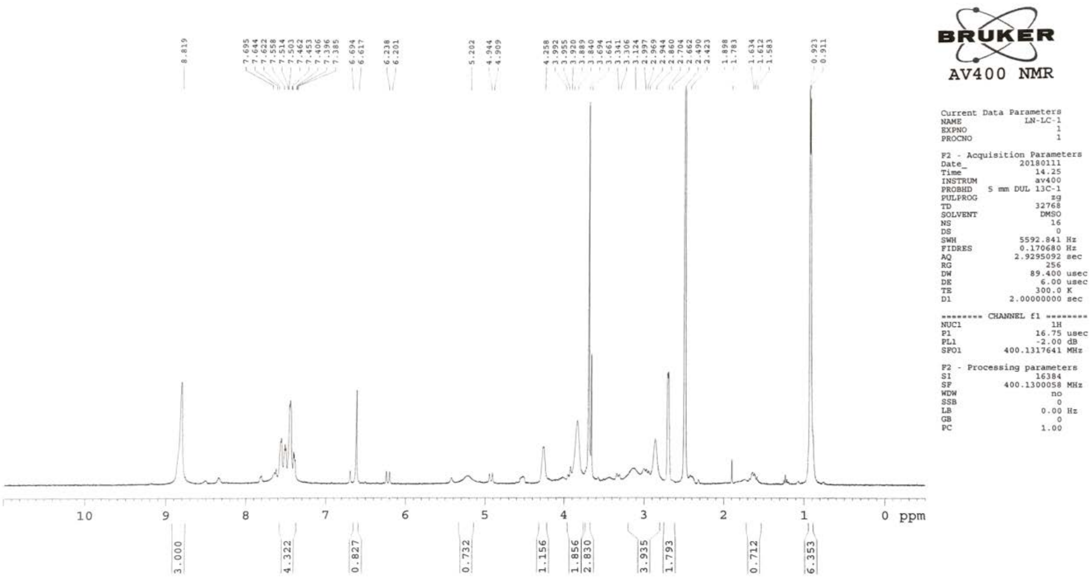

Supplementary Figure 4. ^{1}H-NMR of **5c**.

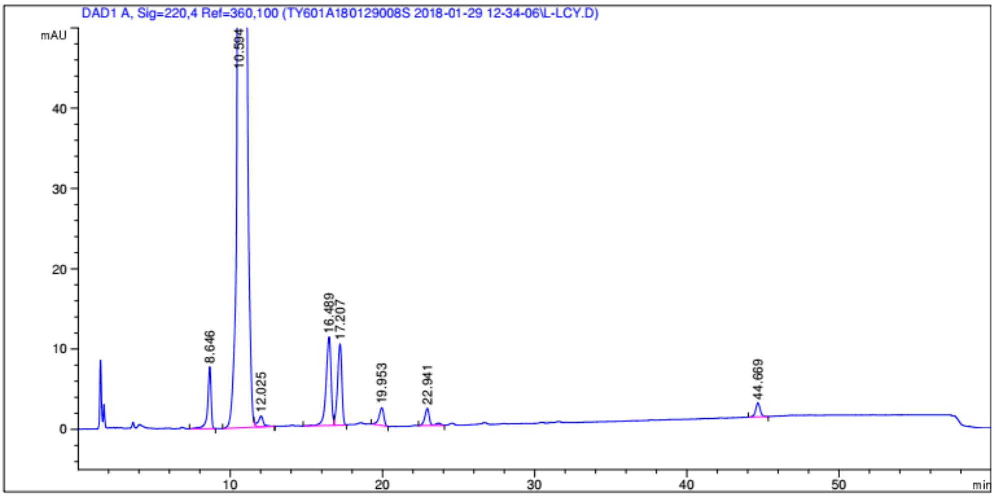

Supplementary Figure 5. HPLC of **5c**.
